# Water Transparency Drives Intra-Population Divergence in Eurasian Perch (*Perca fluviatilis*)

**DOI:** 10.1371/journal.pone.0043641

**Published:** 2012-08-17

**Authors:** Pia Bartels, Philipp E. Hirsch, Richard Svanbäck, Peter Eklöv

**Affiliations:** Department of Ecology and Genetics, Limnology, Uppsala University, Uppsala, Sweden; University of Arkanas, United States of America

## Abstract

Trait combinations that lead to a higher efficiency in resource utilization are important drivers of divergent natural selection and adaptive radiation. However, variation in environmental features might constrain foraging in complex ways and therefore impede the exploitation of critical resources. We tested the effect of water transparency on intra-population divergence in morphology of Eurasian perch (*Perca fluviatilis*) across seven lakes in central Sweden. Morphological divergence between near-shore littoral and open-water pelagic perch substantially increased with increasing water transparency. Reliance on littoral resources increased strongly with increasing water transparency in littoral populations, whereas littoral reliance was not affected by water transparency in pelagic populations. Despite the similar reliance on pelagic resources in pelagic populations along the water transparency gradient, the utilization of particular pelagic prey items differed with variation in water transparency in pelagic populations. Pelagic perch utilized cladocerans in lakes with high water transparency and copepods in lakes with low water transparency. We suggest that under impaired visual conditions low utilization of littoral resources by littoral perch and utilization of evasive copepods by pelagic perch may lead to changes in morphology. Our findings indicate that visual conditions can affect population divergence in predator populations through their effects on resource utilization.

## Introduction

Differences in habitat and resource use have long been viewed as a major cause of phenotypic divergence within and between species. This is because different environments require adaptations of behavioral, morphological, or life history traits [1] which increase the individuaĺs fitness and may ultimately lead to adaptive radiation and ecological speciation [2]. In particular, the exploitation of different resources is thought to drive population divergence through resource polymorphism [2] leading to a correlation between trophic traits and feeding efficiency on specific resources [3], [4]. Trophic polymorphism is common [2] as seen, for example, in the adaptive variation in beak morphology in Darwin’s finches [5] or the feeding morphology in some African cichlids [6].

Aquatic ecosystems are greatly susceptible to environmental change following natural or anthropogenic activities. In particular, water transparency is affected by multiple processes. For instance, elevated sediment loading from the watershed or sediment re-suspension can increase sedimentary turbidity, which is acknowledged as a major environmental problem [7]. During eutrophication, turbidity can also increase due to enhanced phytoplankton growth [8]. Increasing brown coloration due to elevated inputs of dissolved organic matter (DOM) can further decrease water transparency and this “brownification” has been progressively observed in lakes in the Northern Hemisphere [9], [10]. A decrease in water transparency, regardless of the cause (i.e. turbidity or DOM) can affect aquatic organisms that depend on vision for foraging, mating, or intra-specific communication [11]–[13]. Previous studies exploring the consequences of decreased water transparency on population divergence have mainly focused on the weakening effects of low water transparency on the latter two [12], [14]. For instance, Seehausen and coworkers [14] showed that fewer and duller color morphs of Lake Victorian cichlids were found in areas with increased turbidity. However, water transparency might also influence population divergence through its effects on foraging behavior if the divergence is driven by the exploitation of different resources. In lake ecosystems, fish often occupy either near-shore littoral or open-water pelagic habitats resulting in the evolution of habitat-specific traits [2]. Such polymorphism usually takes the form of streamlined individuals foraging in the pelagic zone of lakes whereas deeper bodied individuals forage in the littoral zone [3]. Several studies showed that low water transparency due to high turbidity or water coloration limit the foraging abilities of fish [13], [15], [16], potentially impeding the utilization of critical resources. Furthermore, low water transparency might cause alterations in resource use [17] because some prey types might be more visible than others [18]. Although alterations in resource use or in foraging behavior due to decreasing water transparency have previously been studied their effects on population divergence are currently not known.

Changes in water transparency can additionally affect important properties of aquatic ecosystems that likely influence the exploitation of critical habitats or resources, resulting in variation in phenotypic divergence. Water transparency has repeatedly been shown to affect littoral primary production [19], [20]. For instance, eutrophication has been demonstrated to impede benthic energy pathways through enhanced pelagic productivity [21]. Similarly, decreasing light penetration through the water column due to enhanced input of terrestrial organic matter can hamper benthic productivity [22]. Irrespective of the cause of the reduction, decreased benthic productivity likely limits the amount of benthic resources that can be used by fish. Changes in the availability of critical resources due to alterations in habitat productivity might affect resource exploitation, and thus population divergence.

Eurasian perch (*Perca fluviatilis*) displays a continuous phenotypic variation in relation to habitat and resource use where more streamlined individuals feeding mainly on pelagic resources are found in the pelagic zone and deeper bodied individuals that utilize benthic resources in the littoral zone [23], [24]. Moreover, Ljunggren and Sandström [13] showed that perch forging is strongly affected by water transparency, suggesting that water transparency can indirectly influence morphological divergence through its effect on foraging. Here, we used seven lakes in central Sweden to investigate the effect of water transparency on morphological divergence in perch. Our major objective was to examine whether water transparency affects phenotypic divergence in a predatory fish by modifying its foraging behavior. In particular we investigated two hypotheses and predicted that: i) intra-population divergence would be stronger at high water transparency due to the availability of alternate habitats and resources, and ii) intra-population divergence would be low in lakes with low water transparency due to higher similarity of resource use by pelagic and littoral perch.

## Methods

**Figure 1:**
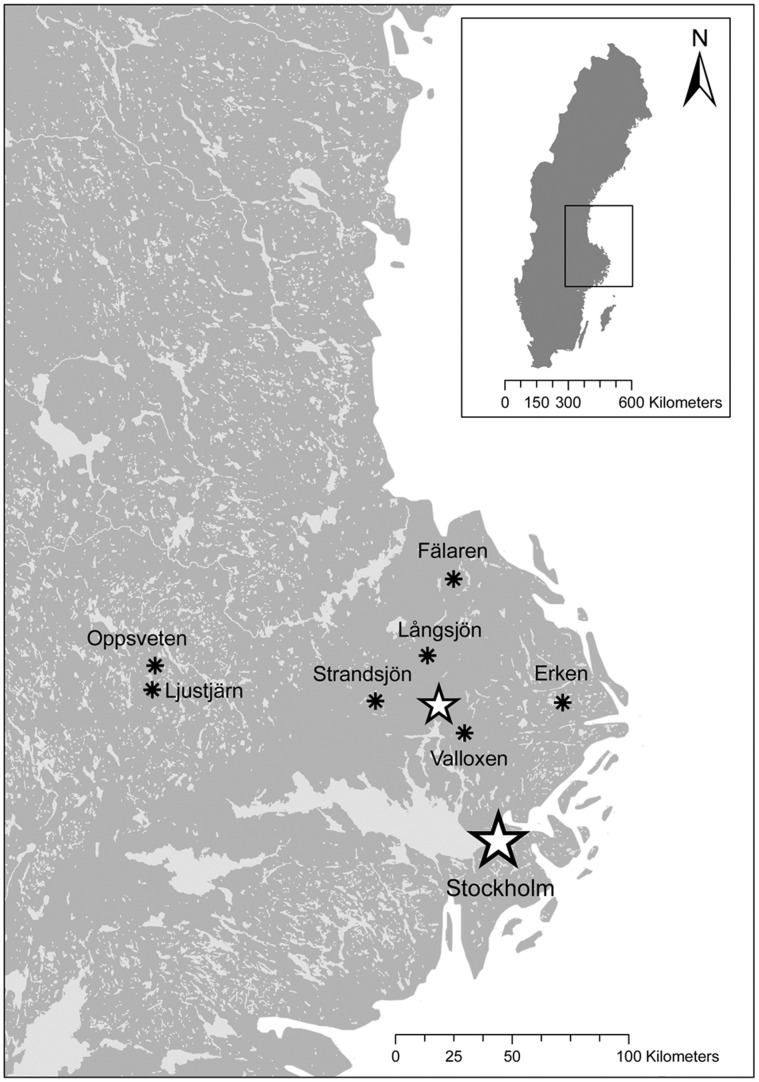
Map of surveyed lakes. Location of the seven lakes included in the field survey. The small star represents the location of Uppsala. Copyright Lantmäteriet Gävle (2010): Permission I 2010/0058.

We conducted a field survey in seven Swedish lakes (Figure 1, Table 1). All necessary permits were obtained for the described field study. No specific permissions were required for five lakes since the lakes were not privately owned or protected. The access of two lakes (Långsjön, Valloxen) was situated on private land, and we obtained access permission from the landowner. For each lake, we calculated the shoreline index as an approximation of the littoral zone [25]. Perimeter and area of each lake were estimated using GIS (ArcGis 9.1, ESRI). Our lake survey contained both turbid (n = 1) and brown-colored humic (n = 3) lakes therefore we used water transparency as a comparable measurement for visual conditions among lakes. Water transparency is a measure of how clear or transparent the water is and depends on both watercolor and light scattering. We used Secchi depth as it is one of the most commonly used tools to measure water transparency [26]. We used standardized multi-mesh gill nets (littoral nets: 30×1.5 m; pelagic nets: 27.5×6 m) to estimate relative biomass and species composition in the littoral and pelagic habitats of each lake. Fish were measured to the nearest 1 mm (total length), weighed to the nearest 0.1 g, and stored frozen at −20°C until further analyses. To estimate the condition of fish, we calculated the Fulton’s condition factor (weight × length**^−^** ^3^).

**Table 1:** Main characteristics of studied lakes in central Sweden.

	Ljustjärn	Långsjön	Erken	Oppsveten	Strandsjön	Fälaren	Valloxen
**Location**	N59°55′ E15°26′	N60°01′ E17°34′	N59°50′ E18°33′	N60°00′ E15°25′	N59°52′ E17°09′	N60°20′ E17°47″	N59°44′ E17°50′
**Sampling date**	08/2007	08/2008	08/2008	08/2007	08/2008	08/2007	08/2008
**Area (km^2^)**	0.12	2.5	23.7	0.65	1.3	2.05	2.9
**Max depth (m)**	11	12.5	21.0	10	4.0	2.6	9.0
**Average depth (m)**	–	6.3	9.0	–	1.7	1.5	3.8
**Tot-P (µg L^−1^)**	12.1	17.0	27.0	15.4	15.4	20.5	46.7
**DOC (mg L^−1^)**	5.9	6.2	10.3	19.1	20.8	34.3	18.9
**Secchi depth (m)**	5.7	5.6	5.4	1.8	1.6	1.5	1.1
**Shoreline index**	2.65	3.77	5.54	2.52	3.51	2.89	5.28

Values represent summer measurements from one sampling occasion.

Zooplankton were sampled with a 100 µm-mesh net (Ø 25 cm) and samples were preserved with Lugol’s solution. In the littoral zone, the net was towed horizontally for approximately 2 m parallel to the shoreline, whereas in the pelagic zone, one vertical tow was made at the deepest point from approximately 1 m above the sediment to the surface. Three samples were taken each in the littoral and pelagic habitat to account for spatial variability. Zooplankton were counted, measured, and identified. Individuals were categorized into (1) cladocerans, (2) copepods, and (3) other (mainly *Chaoborus* sp. and rotifers). Biomass was calculated using published mass-length relationships [27].

Macrozoobenthos was sampled with a core sampler (Ø 60 mm, UWITEC®, Vienna, Austria). Four samples were taken to account for potential spatial variability in the littoral (approx. 1 m depth) and the profundal zone (approx. at maximum depth). Only the upper 5 cm of the sediment were used for analyses. Samples were sieved using a 0.5-mm net, preserved in 70% ethanol and stained with Bengal rose. In the laboratory, macroinvertebrates were counted, measured, and identified to lowest possible taxonomic level. Biomass (dry weight) was calculated using published mass-length relationships [28]–[30]. The field study did not involve endangered or protected species.

To evaluate the effect of water transparency and lake depth on resource composition, we used permutational multivariate analysis of variance (“perm MANOVA”; ref [31]) with Secchi depth or maximum lake depth as predictors and littoral and pelagic resource composition, respectively, as response variables.

### Morphological Analyses

**Figure 2:**
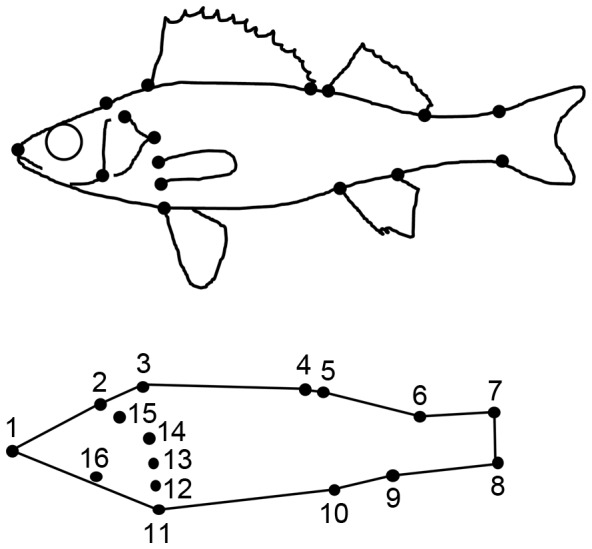
Position of landmarks. Location of the 16 landmarks used in morphological analyses.

Perch morphology was analyzed using landmark-based thin-plate spline (TPS) analysis, a geometric morphometrics technique [32]. We used the programs TPS-dig2, TPS-relw, and TPS-regr for all morphological analyses (available at http://life.bio.sunysb.edu/morph/index.html). All fish were thawed and photographed on the left side. Subsequently, we analyzed the morphology using 16 landmarks digitized with TPS-dig2 for each image (Figure 2). Perch morphology was analyzed in the same way as we have described previously [23], [33]. In short, we used the digitized landmarks to analyze the relative position of each landmark and variation in body shape using TPS-relw by calculating partial warps and uniform scores for each individual [34]. TPS-relw transforms all specimens to a centroid size to avoid differences in landmarks due to body size. The uniform shape components parameterize all shape variation that is uniform throughout the whole geometry. The partial warps measure non-uniform shape variation that is localized to particular regions of the geometry. The differences in morphology of perch were analyzed among lakes and between littoral and pelagic habitats separately for each lake. The partial warps and uniform scores were analyzed with a discriminant function analysis (DFA). The DFA combines all partial warps and uniform scores for each fish into n-1 functions (morphological indices) that maximally discriminate between the groups where n is the number of classification levels. To compare perch morphology across lakes, we combined the partial warps and uniform scores from all fish in one DFA and based the classification on lakes (n = 7). To compare perch morphology between habitats, we analyzed the partial warps and uniform scores separately for each lake and based the classification in the DFA on habitat (n = 2). TPS-regr was used to visualize the differences in body shape of perch [35]. To test for differences between habitats, we used linear mixed effect models (LME) with the DFA scores as response variable, habitat as fixed effect and lake nested in habitat as random effect.

Morphological divergence was calculated as the difference between mean littoral and mean pelagic morphological index derived from the separate DFA analyses for each lake. To evaluate the most important factors affecting morphological divergence, we used PLS (partial least squares regression analysis; ref [36]). PLS is the preferred method for constructing models when predictors are many and highly collinear [36]. R^2^Y and Q^2^ were used to evaluate our model. R^2^Y is comparable to the R^2^ in linear regressions. Q^2^ is a measure of the predictive power of the model (the closer Q^2^ is to R^2^Y, the higher the predictive power). We used the variable influence on projection (VIP) to estimate the influence of every X-variable on the Y-component (morphological divergence; Table S1). The VIP scores for every X-variable are cumulative across components and weighted according to the amount of Y-variance explained by each component [36]. X-variables with VIP>1 were considered most influential for the models. To evaluate the relationship between morphological divergence and predictors with VIP>1, we used regression models. For each model, we calculated the second-order or small sample Akaike’s information criterion (AICc; ref [37], [38]) as an estimation of model fit which corrects for sample size and model complexity [39]. In the model selection, we tested each predictor independently with increasing model complexity (i.e. from a linear to a polynomial model). This was done until AICc reached minimal values. A model was considered more likely when ΔAICc ≥ 4 [39].

### Stable Isotope Analyses

We used stable isotope analyses to estimate the resource utilization from littoral and pelagic habitats. Stable isotopes integrate resource use over longer time periods (approximately 1.5 months in perch; ref [24]). In addition, pelagic and littoral resources are easily distinguishable in most cases as pelagic resources are more depleted in δ^13^C than littoral resources. In each lake, we collected snails (*Lymnea stagnalis*, *Radix balthica*) from the littoral zone and zooplankton from the pelagic zone in order to obtain a baseline signature for δ^13^C and δ^15^N. Zooplankton were collected by vertically towing a 100 µm-mesh net (Ø 25 cm) multiple times at the deepest points of each lake, from approximately 1 m above the sediment to the surface. Samples were then filtered on GF/F filters. We were not able to collect zooplankton in one of the lakes. Here, we used mussels (*Anodonta* sp.) instead to obtain the pelagic baseline signature. Snails and mussels were kept alive for 48 hours in GF/F filtered lake water to enable gut evacuation and subsequently removed from their shells. Dorsal muscle tissue was sampled from up to 30 littoral and 30 pelagic randomly chosen perch from each lake. All animal tissue was dried (60°C for 48°hours), ground and stored in tin capsules. Muscle tissue from perch was not corrected for lipid content due to its low average C:N ratio [40], [41].

Stable isotope analyses were carried out at the University of California, Davis Stable Isotope Facility, California, on a continuous-flow isotope ratio mass spectrometer (PDZ Europa 20–20). The results are expressed using the delta (δ) notation in ‰ as δ = (R_sample_:R_standard_ –1)×1000, where R = ^13^C:^12^C or ^15^N:^14^N. Standards used were Pee Dee belemnite (PDB) for δ^13^C and atmospheric nitrogen for δ^15^N. A quarter of the samples were analyzed in triplicate and the analytical error was 0.04‰ and 0.21‰ for δ^13^C and δ^15^N, respectively.

We used IsoError 1.04 [42] to estimate the contribution of littoral resources to perch diet. IsoError uses linear mixing models to quantify the contribution of two sources (i.e., pelagic and littoral resources) to a mixture (i.e., fish diet). For each lake, we used the lake-specific littoral and pelagic resources to calculate their contribution to perch diet. Prior to the calculations, littoral and pelagic resources were corrected for trophic fractionation using a fractionation factor of 0.47‰ for δ^13^C [43]. We assumed a trophic position of 2 for snails, mussels, and zooplankton.

### Stomach Content Analyses

The stomach content of fish was analyzed under a dissection microscope and the food items were separated into six diet categories, (1) benthos, (2) cladocerans, (3) copepods, (4) pelagic macroinvertebrates, (5) terrestrial prey, and (6) fish. The lengths of 10 prey of each group were measured to the nearest 0.1 mm. In groups of <10 individuals, all prey were measured. Average lengths were then used to calculate biomass (dry weight) for all prey types. Pelagic macroinvertebrates consisted of chironomid pupae and *Chaoborus* sp. larvae. Only 23 from a total of 1135 perch contained terrestrial prey or fish in their diet, and therefore these categories were excluded from statistical analyses. The proportion of perch with empty stomachs was only marginally affected by water transparency (generalized linear model: p = 0.09), and therefore perch with empty stomachs were excluded from further diet analyses. To test for differences in littoral resource use between habitats, we used linear mixed effect models (LME) with the contribution of benthos to perch diet as response variable, habitat as fixed effect and lake nested in habitat as random effect.

To quantify the diet overlap between littoral and pelagic perch populations, we used Schoener’s similarity index (S) [44]:
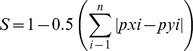
where *p_xi_* is the mean proportion of food category *i* in the diet of littoral perch, *p_yi_* is the mean proportion of food category *i* in the diet of pelagic perch and *n* equals the number of food categories. Values of *S* approach 0 for populations that share no prey types and 1 for populations that have completely identical prey utilization. A diet overlap of more than 60% (S >0.6) was considered as a substantial overlap [45].

PLS modeling was done in SIMCA 12.0 (Umetrics AB, Umeå, Sweden). All other analyses were performed in R 2.15.

## Results

Fish communities in all lakes were dominated by roach (*Rutilus rutilus*) and perch (Table S2). Other common species were bleak (*Alburnus alburnus*), common bream (*Abramis brama*), smelt (*Osmerus eperlanus*), tench (*Tinca tinca*), white bream (*Blicca bjoerkna*), rudd (*Scardinius erythrophthalmus*), ruffe (*Gymnocephalus cernuus*), northern pike (*Esox lucius*), and pikeperch (*Sander lucioperca*).

### Resource Availability

Total biomass of benthic and pelagic resources in littoral and pelagic habitats (Table S3) was not affected by water transparency (Pearson correlation: r = −0.06 to 0.27, p>0.56). Water transparency and lake depth did not affect pelagic and littoral resource composition (perm MANOVA: water transparency: zooplankton: p = 0.11; benthos: p = 0.67; lake depth: zooplankton: p = 0.42; benthos: p = 0.45).

### Morphological Analysis

**Figure 3:**
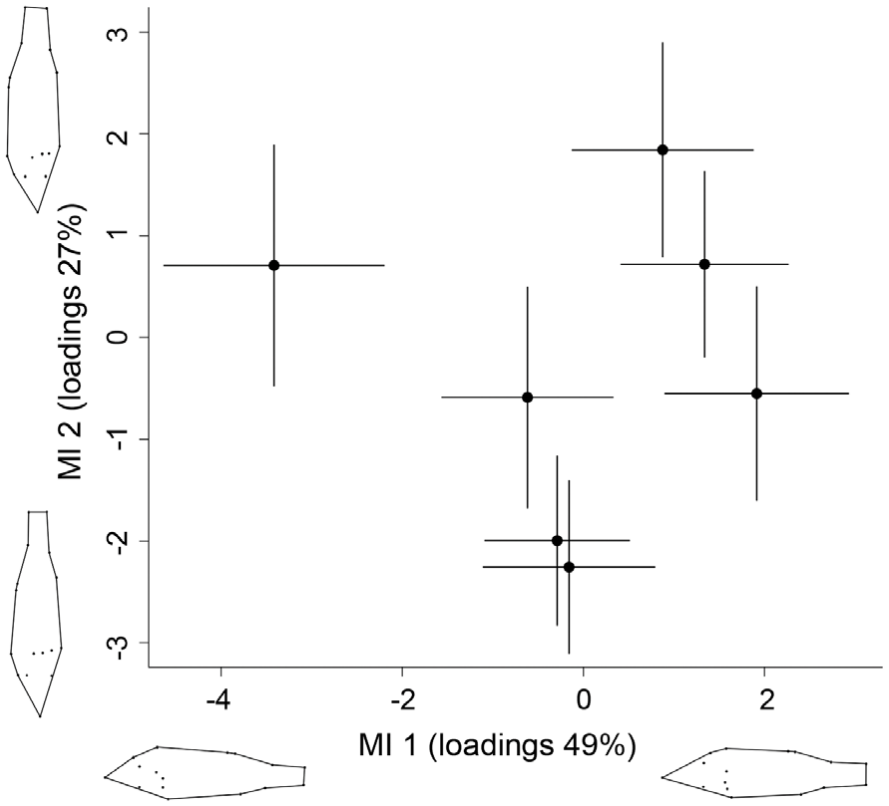
Morphological variation across lakes. Variation (mean ± 1SD) along the first (MI1) and second (MI 2) morphological axis across all surveyed lakes. Deformation plots (uniform and non-uniform components) corresponding to variation in MI 1 and MI 2 are shown beside the axes.

The DFA analysis of body morphology across all lakes revealed 2 significant morphological axes (p<0.05; Figure 3). The score values of the first morphological axis, explaining variation in head morphology, decreased with increasing water transparency (r = −0.56, p = 0.19), whereas the score values of the second morphological axis, explaining variation in body depth, increased with increasing water transparency (r = 0.57, p = 0.18). However, none of the correlations were significant, meaning there were no gross morphological differences across lakes.

**Figure 4:**
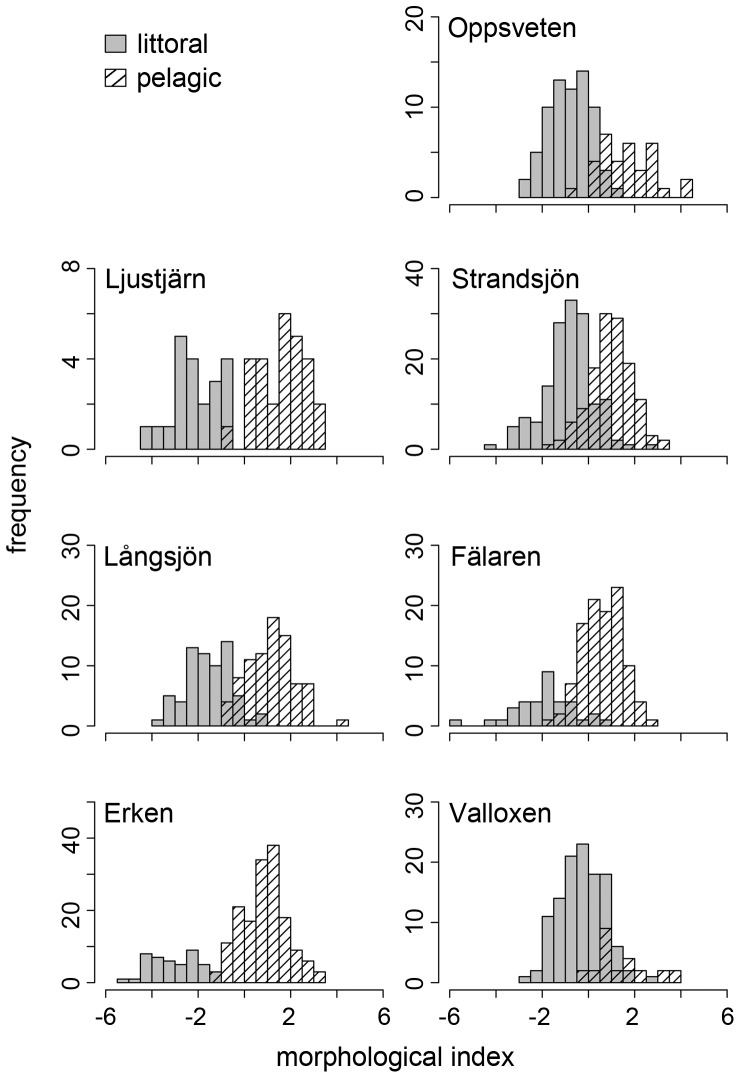
Morphology of littoral and pelagic perch. Frequency distribution of perch DFA morphological scores from surveyed lakes.

Perch morphology (DFA scores) differed between littoral and pelagic habitats in all lakes (LME: p<0.001) with generally deeper-bodied perch in the littoral zone, and more streamlined perch occupying the pelagic zone. Morphological divergence between littoral and pelagic perch however, differed between the surveyed lakes (Figure 4). To evaluate which predictors were most influential on morphological divergence we used PLS analysis. The PLS regression model explained in total 82% of the variance (R^2^Y_cum_ = 0.82) and the model predictability was moderately high (Q^2^_cum_ = 0.43). Based on the VIP scores (VIP>1), the most important predictors of morphological divergence included, with decreasing importance, the proportion of cladocerans to perch diet, Secchi depth, catch per unit effort (CPUE) of roach in the littoral zone, condition factor, the contribution of copepods to perch diet, piscivore CPUE in the littoral, maximum depth, growth rate, and dissolved organic carbon (Figure S1).

Individual linear regression models revealed that morphological divergence increased with increasing condition factor, contribution of cladocerans to perch diet, growth rate, piscivore CPUE in the littoral, maximum depth, and Secchi depth, whereas it decreased with increasing dissolved organic carbon and CPUE of roach in the littoral (Table 2). The relationship between morphological divergence and contribution of copepods to perch diet was best described with a polynomial function (Table 2). However, only the contribution of cladocerans and copepods to perch diet, CPUE of roach in the littoral, and Secchi depth were significantly correlated with morphological divergence (Table 2), and the AICc suggested no difference in fit between the four models. Water transparency explained most of the variance (70%), whereas contribution of cladocerans and copepods to perch diet and CPUE of roach in the littoral explained 68%, 67%, and 57%, respectively (Table 2).

**Table 2:** Summary of model selection.

Response [y]	Predictor [x]	Model	Direction	k	df	AICc	adj. R^2^	p
Morphological divergence	cf	y = a+bx	positive	3	4	25.43	0.46	0.057
	**copep**	**y = a+bx+cx^2^**	**–**	**4**	**3**	**23.42**	**0.67**	**0.049**
	**clad**	**y = a+bx**	**positive**	**3**	**4**	**21.64**	**0.68**	**0.013**
	log(doc)	y = a+bx	negative	3	4	27.43	0.28	0.13
	log(growth)	y = a+bx	positive	3	4	27.93	0.22	0.16
	**roach.lit**	**y = a+bx**	**negative**	**3**	**4**	**23.84**	**0.57**	**0.030**
	log(pisc.lit)	y = a+bx	positive	3	4	28.70	0.13	0.22
	depth	y = a+bx	positive	3	4	26.42	0.37	0.085
	**log(secchi)**	**y = a+bx**	**positive**	**3**	**4**	**21.37**	**0.70**	**0.012**

Given are the predictor and the response variables with VIP>1 in PLS included in each model, the model equations (Model), the direction of the relationship (Direction), number of parameters included in each model (k), degrees of freedom (df), second-order Akaike’s information criterion (AICc) as an estimation of model fit, the adjusted R^2^ and the p-value. cf = condition factor, copep = contribution of copepods to diet, clad = contribution of cladocerans to diet, roach.lit = littoral CPUE of roach, pisc.lit = littoral CPUE of piscivores.

### Stable Isotope Analysis

**Figure 5:**
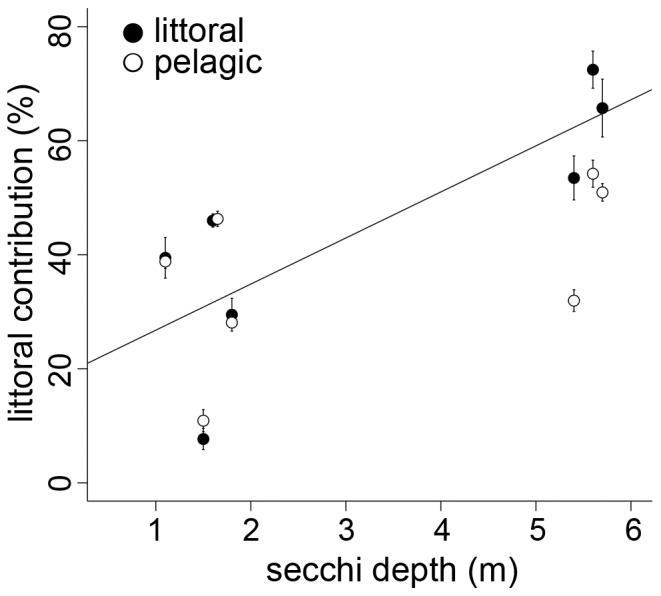
Littoral reliance of littoral and pelagic perch. Littoral reliance (mean ± 1SE) as a function of Secchi depth for littoral and pelagic perch populations. Regression line drawn for littoral perch populations.

The reliance on littoral resources inferred from stable isotopes analysis varied among lake ecosystems, ranging between 9.6±10.9% and 63.3±18%. Generally, littoral reliance for the whole population tended to increase with increasing water transparency (r = 0.70, p = 0.080), whereas the shoreline index and lake depth did not affect littoral reliance (r = 0.18, p = 0.71 and r = 0.45, p = 0.31, respectively). However, in separate analyses, littoral reliance was not affected by water transparency in pelagic populations (range: 10.9±3.6% to 54.2±13.0%, r = 0.52, p = 0.23; Figure 5), whereas littoral populations increasingly relied on littoral resources with increasing water transparency (range: 7.7±9.1% to 72.5±17.9%, r = 0.80, p = 0.031; Figure 5).

### Stomach Content Analysis

**Figure 6:**
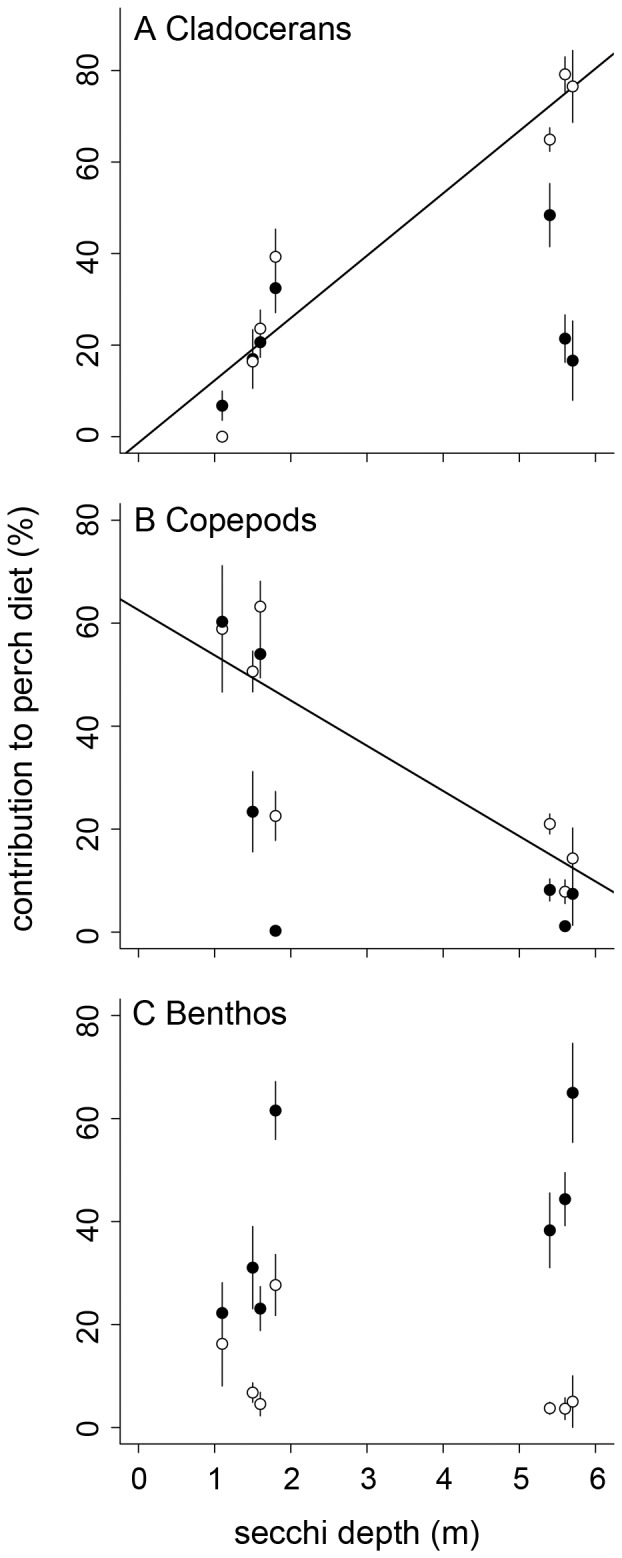
Diet contribution to littoral and pelagic perch. Contribution (mean ±1 SE) of resources to perch stomach content as a function of Secchi depth. Open symbols = pelagic perch, solid symbols = littoral perch. Regression line (A, B) shown for pelagic perch populations.

Generally, littoral perch consumed higher proportions of benthic resources than pelagic perch (mean ±1 SD: littoral: 38.4±43.0; pelagic: 6.6±20.4; LME: p<0.01; Figure S2). However, the diet overlap between littoral and pelagic perch varied substantially with variation in water transparency (range: 32–83%) and decreased with increasing water transparency (r = −0.78, p = 0.040). The contribution of cladocerans, copepods, and benthos to littoral perch diet was not affected by water transparency (cladocerans: r = 0.42, p = 0.35; copepods: r = −0.61, p = 0.15; benthos: r = 0.53, p = 0.22; Figure 6). In contrast, the contribution of cladocerans to pelagic perch diet increased strongly with increasing water transparency (r = 0.90, p = 0.006; Figure 6A), whereas the contribution of copepods decreased with increasing water transparency (r = −0.85, p = 0.015; Figure 6B). The contribution of benthos to pelagic perch was not affected by water transparency (r = −0.59, p = 0.16; Figure 6C).

## Discussion

In our study, water transparency was identified as an important predictor of population divergence in a predatory fish. Furthermore, we identified diet composition and CPUE of roach in the littoral as additional factors influencing population divergence thus indicating that foraging behavior and interspecific competition also play an important role in population divergence. Water transparency mediates several processes in aquatic ecosystems. In particular, changes in water transparency can affect foraging behavior and competitive interactions. Below we elaborate on potential mechanisms explaining the observed patterns in population divergence.

### Foraging Behavior and Resource Availability

Differences in habitat and resource use are major determinants of population divergence. Different resources may require specific trait adaptations that lead to increased performance such as the variation in beak morphology in Darwin’s finches in response to differences in size and hardness of seeds [5]. In fish, pelagic individuals are usually more streamlined to increase long distance swimming performance and to facilitate feeding on widely dispersed prey. Littoral individuals, on the other hand, tend to be deeper-bodied, optimally adjusted to maneuver in structural complex environments, and to feed on benthic prey [3], [4]. If the utilization of critical resources is limited, this might impede the evolution of trait adaptations. Though it has been suggested that benthic foraging is less vision-dependent than pelagic foraging [46], several studies show that low water transparency can impair benthic forging efficiency [47], [48]. In our study, littoral perch increased the utilization of littoral resources estimated by stable isotope analysis with increasing water transparency. As the abundance of littoral resources was similar along the water transparency gradient, this suggests that benthic foraging was likely reduced in low water transparency. However, the contribution of littoral resources to perch diet inferred from stomach content analysis was similar along the water transparency gradient. Stable isotope analyses integrate resource utilization over long term (typically 1.5 months in perch; ref [24]), and are therefore a better estimate of resource use than stomach content analysis. Littoral resource productivity and abundance of littoral prey vary temporarily [49], [50], but are typically high in summer. Benthic foraging is likely less affected by low water transparency when resource availability is high, but might be substantially affected at low resource availability. Furthermore, resource availability might be lower in lakes with low water transparency at other times, though we did not assess that. Previous studies reported that decreasing water transparency due to eutrophication or increasing input of terrestrial organic matter can limit littoral productivity due to light limitation, resulting in a higher relative importance of pelagic productivity and reduced littoral reliance of fish [21], [22]. It is likely a combination of reduced benthic foraging efficiency and limited littoral resource availability that resulted in decreasing reliance on littoral resources in perch.

The reliance on pelagic resources of pelagic perch was similar across the water transparency gradient, however the acquisition of particular pelagic prey items differed substantially. With increasing water transparency, the consumption of cladocerans increased, whereas the consumption of copepods decreased. Similarly, Estlander et al. [15] reported that selectivity of perch for daphnids decreased in brown-colored water. In low water transparency, prey motion might be more important than size or visibility. Cladocerans generally move slowly whereas copepods are capable of evasive movements [51]. The fast and irregular movements of copepods might make them better detectable when water transparency is low and thus more susceptible to perch predation. A previous study reported that bluegill (*Lepomis macrochirus*) preferred faster swimming clones of *Daphnia* [52], supporting the idea that prey motion increases conspicuousness to fish and thus the effectiveness of prey detection. Limited utilization of littoral resources and differences in diet composition of pelagic prey resulted in increasing similarity of resource utilization of littoral and pelagic perch with decreasing water transparency, and thus decreasing population divergence.

### Interspecific Competition

The competitive interaction between roach and perch has been demonstrated in numerous studies as asymmetric with roach being the superior competitor for zooplankton [53]. Moreover, perch coexisting with roach have been shown to feed on copepods, whereas in the absence of roach, perch mainly fed on cladocerans [54]. Roach abundance in the littoral habitat was similar across the water transparency gradient (Table S4), suggesting that interspecific competition per se did not result in the observed switch of feeding on cladocerans to feeding on copepods in perch. However, water transparency can alter competitive interactions between roach and perch. Estlander et al. [15] showed that selectivity of perch for daphnids decreased with decreasing water transparency, whereas selectivity of roach was not affected by changes in water transparency, suggesting that decreasing water transparency resulted in a competitive advantage of roach.

### Caveat and Future Directions

Our study identified the most important predictors of population divergence between littoral and pelagic perch. Although we present several potential mechanisms, our study cannot provide the ultimate causes for the observed patterns. For instance, diet composition of perch and interspecific competition played a major role in determining population divergence, however the extent to which each factor contributed to differences in population divergence and potential interactions remain to be investigated. Future studies should explicitly test the underlying mechanisms to disentangle the contribution of each factor to differences in population divergence.

Most studies investigating the effects of water transparency on fish foraging used turbidity, less is known about the effects of variation in watercolor (driven by increased DOM) on fish foraging. The optical characteristics differ substantially between turbid and brown-colored water. In turbid water, suspended material such as sediment particles or phytoplankton, scatter and attenuate incoming light, independent of wavelength. In contrast, the brown color originating from humic substances selectively absorbs short wavelengths of incoming light and therefore results in a wavelength shift of maximum transmission towards longer wavelengths [55], [56]. While watercolor mainly reduces light intensity [57], [58], turbidity can change the contrast between an object and the background [59]. A reduction in contrast between prey and its background might limit the detection by a predator even if light levels are sufficient. To our knowledge, only one study compared differences in foraging behavior of fish between turbid and brown-colored water [16]. However the authors found that reaction distance and attack rate were similar in turbid and brown-water treatments, suggesting that turbidity and watercolor have similar effects on foraging behavior. In our study, most lakes with low water transparency were humic lakes, whereas only one lake was potentially more turbid. Future studies should focus on the differences of turbidity and watercolor on fish foraging behavior.

### Conclusions

In conclusion, our study shows that water transparency plays an important role in influencing population divergence in an aquatic organism. In aquatic ecosystems, water transparency is greatly impacted by anthropogenic activities, and can be impaired by several factors such as increased turbidity due to eutrophication or high sediment loads, or altered watercolor due to the input of terrestrial organic matter [7], [9], [60]. Increasing eutrophication has resulted in the loss of a stickleback species pair [61] and has affected mating systems in cichlids where fewer and duller color morphs were found in areas that experienced human-induced increased turbidity [14]. We suggest that changes in water transparency, independent of their cause, can also influence population divergence through their effects on resource utilization.

## Supporting Information

Figure S1 **VIP scores of PLS analysis identifying the main factors related to morphological divergence.** VIP is normalized, the average squared VIP value is 1. Terms in the model with a VIP>1 are important.
(TIF)

Figure S2 **Diet composition (%) of perch stomach content from A) littoral and B) pelagic fish.** Other = *Chaoborus* sp., Rotatoria, fish, and terrestrial prey.
(TIF)

Table S1 **Variables used in the PLS analysis.**
(DOCX)

Table S2 **Catch per unit effort (g m^−2^ net) for all surveyed lakes.**
(DOCX)

Table S3 **Biomass of pelagic (mg L^−1^) and benthic (mg m^−2^) resources for all surveyed lakes.**
(DOCX)

Table S4 **Correlation matrix of predictor variables with VIP>1.** Shown are Pearson’s correlation coefficients. Significance levels *p<0.05, **p<0.01.
(DOCX)
