# Supplementary figures and images for: Water Transparency Drives Intra-Population Divergence in Eurasian Perch (Perca fluviatilis)
Source: PLoS One. 2012 Aug 17;7(8):e43641. doi: 10.1371/journal.pone.0043641 (PMC3422328; doi:10.1371/journal.pone.0043641)

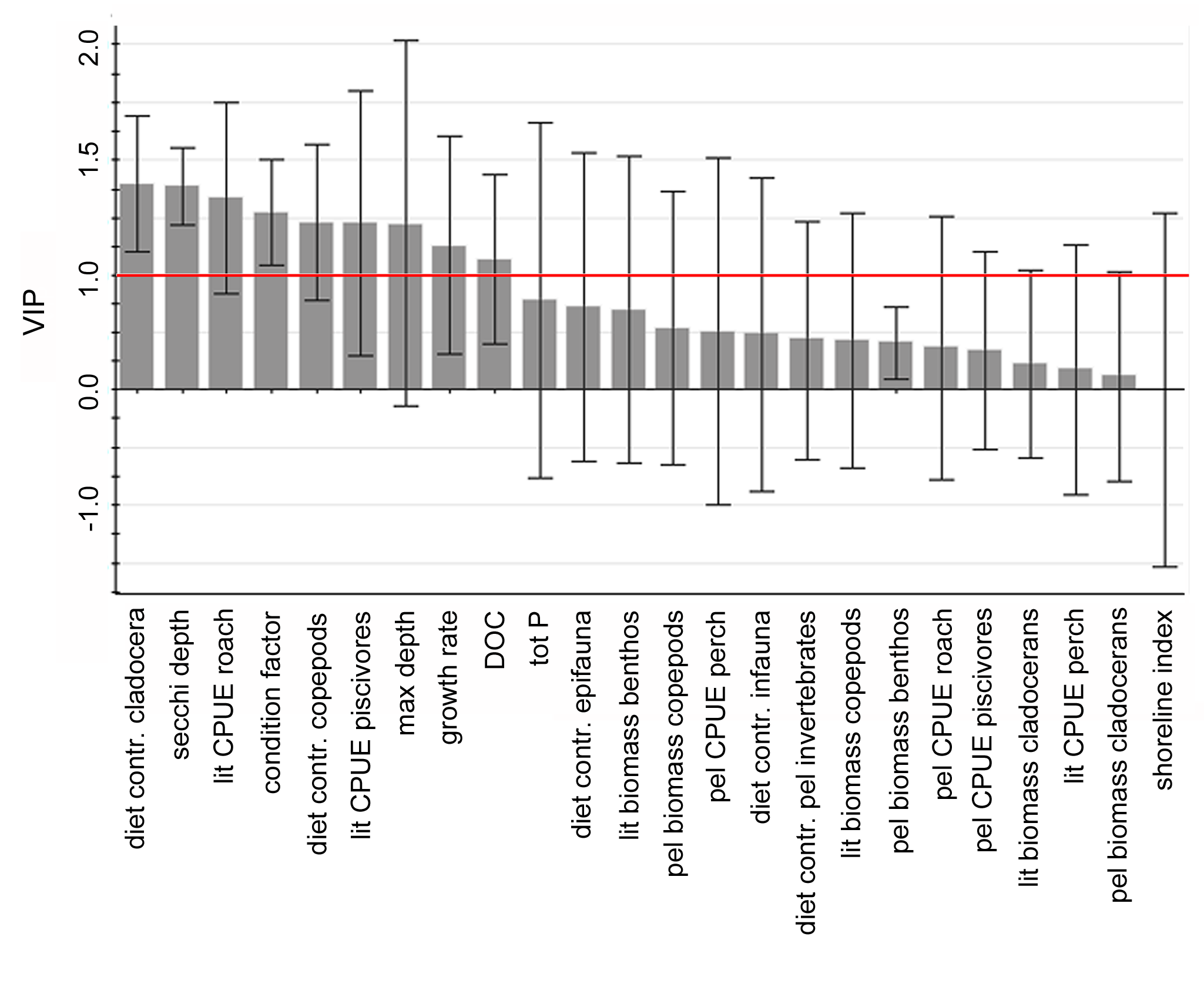

Supplement: Figure S1 — VIP scores of PLS analysis identifying the main factors related to morphological divergence. VIP is normalized, the average squared VIP value is 1. Terms in the model with a VIP>1 are important. (TIF) [file pone.0043641.s001.tif]

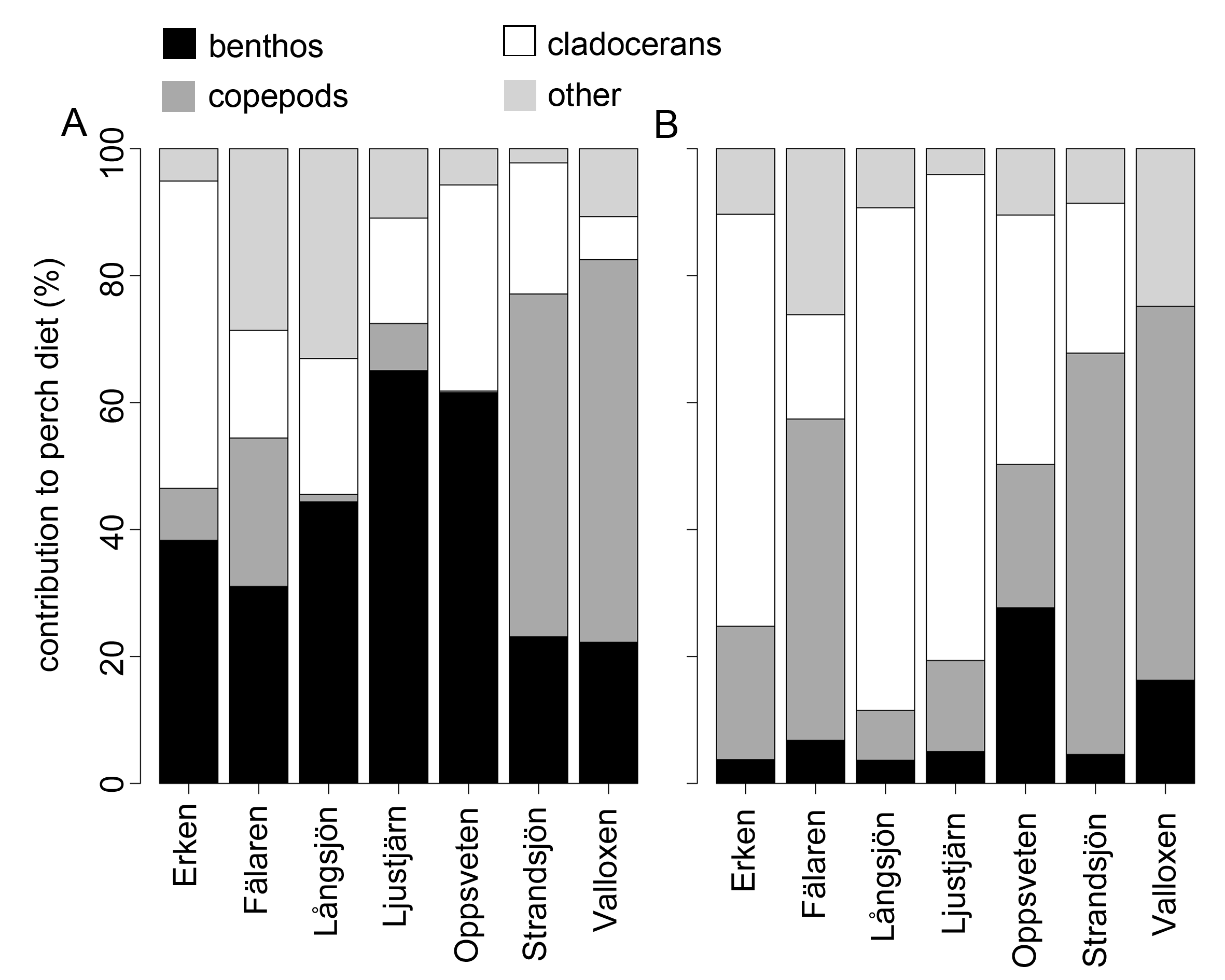

Supplement: Figure S2 — Diet composition (%) of perch stomach content from A) littoral and B) pelagic fish. Other = Chaoborus sp., Rotatoria, fish, and terrestrial prey. (TIF) [file pone.0043641.s002.tif]
