# Supplementary material for: Water Transparency Drives Intra-Population Divergence in Eurasian Perch (Perca fluviatilis)
Source: PLoS One. 2012 Aug 17;7(8):e43641. doi: 10.1371/journal.pone.0043641 (PMC3422328; doi:10.1371/journal.pone.0043641)
Supplement: Table S1 — Variables used in the PLS analysis. (DOCX) [file pone.0043641.s003.docx]

Table S1

| Variable in PLS model | Description | Category |
| --- | --- | --- |
| divergence | morphological divergence | Y |
| condition factor | condition factor | X |
| diet contr. infauna | contribution of chironomids to perch diet (%) | X |
| lit biomass cladocerans | littoral cladoceran biomass (mg L^-1^) | X |
| pel biomass cladocerans | pelagic cladoceran biomass (mg L^-1^) | X |
| lit biomass copepods | littoral copepod biomass (mg L^-1^) | X |
| pel biomass copepods | pelagic copepod biomass (mg L^-1^) | X |
| diet contr. copepods | contribution of copepods to perch diet (%) | X |
| max depth | max depth (m) | X |
| DOC | dissolved organic carbon (mg L^-1^) | X |
| diet contr. epifauna | contribution of epifauna to perch diet (%) | X |
| growth rate | perch growth rate (mm year^-1^) | X |
| lit biomass benthos | littoral macrozoobenthos biomass (mg m^-2^) | X |
| pel biomass benthos | pelagic macrozoobenthos biomass (mg m^-2^) | X |
| diet contr. cladocerans | contribution of cladocerans to perch diet (%) | X |
| diet contr. pel invertebrate | contribution of pelagic invertebrates to perch diet (%) | X |
| lit CPUE perch | CPUE (g m^-2^ net) of perch in littoral | X |
| pel CPUE perch | CPUE (g m^-2^ net) of perch in pelagic | X |
| lit CPUE piscivores | CPUE (g m^-2^ net) of piscivores in littoral | X |
| pel CPUE piscivores | CPUE (g m^-2^ net) of piscivores in pelagic | X |
| lit CPUE roach | CPUE (g m^-2^ net) of roach in littoral | X |
| pel CPUE roach | CPUE (g m^-2^ net) of roach in pelagic | X |
| secchi depth | secchi depth (m) | X |
| shoreline index | shoreline index | X |
| tot P | total phosphorus (µg L^-1^) | X |
