# Supplementary material for: Water Transparency Drives Intra-Population Divergence in Eurasian Perch (Perca fluviatilis)
Source: PLoS One. 2012 Aug 17;7(8):e43641. doi: 10.1371/journal.pone.0043641 (PMC3422328; doi:10.1371/journal.pone.0043641)
Supplement: Table S2 — Catch per unit effort (g m−2 net) for all surveyed lakes. (DOCX) [file pone.0043641.s004.docx]

Table S2

|  | **Ljustjärn** | | **Erken** | | **Långsjön** | | **Oppsveten** | | **Strandsjön** | | **Fälaren** | | **Valloxen** | |
| --- | --- | --- | --- | --- | --- | --- | --- | --- | --- | --- | --- | --- | --- | --- |
|  | *Lit* | *Pel* | *Lit* | *Pel* | *Lit* | *Pel* | *Lit* | *Pel* | *Lit* | *Pel* | *Lit* | *Pel* | *Lit* | *Pel* |
| **total CPUE** | 19.7 | 12.7 | 52.7 | 11.6 | 62.9 | 22.7 | 22.3 | 2.4 | 59.1 | 27.4 | 27.3 | 21.1 | 57.9 | 14.5 |
| **perch** | 4.3 | 3.4 | 12.8 | 7.6 | 4.7 | 6.6 | 15.3 | 1.4 | 12.2 | 8.1 | 5.4 | 6.3 | 6.6 | 0.7 |
| **roach** | 5.7 | 9.3 | 6.8 | 3.6 | 25.0 | 12.1 | 6.9 | 1.0 | 44.4 | 15.9 | 17.1 | 6.0 | 34.1 | 4.5 |
| **piscivores** | 12.6 | 2.4 | 6.2 | 3.5 | 0.5 | 4.4 | 7.2 | 0.1 | 1.9 | 5.8 | 3.7 | 9.8 | 1.9 | 2.7 |
