# Supplementary material for: Water Transparency Drives Intra-Population Divergence in Eurasian Perch (Perca fluviatilis)
Source: PLoS One. 2012 Aug 17;7(8):e43641. doi: 10.1371/journal.pone.0043641 (PMC3422328; doi:10.1371/journal.pone.0043641)
Supplement: Table S3 — Biomass of pelagic (mg L−1) and benthic (mg m−2) resources for all surveyed lakes. (DOCX) [file pone.0043641.s005.docx]

Table S3

|  | |  | **Ljustjärn** | | **Erken** | | **Långsjön** | | **Oppsveten** | | **Strandsjön** | | **Fälaren** | | **Valloxen** | |
| --- | --- | --- | --- | --- | --- | --- | --- | --- | --- | --- | --- | --- | --- | --- | --- | --- |
|  | |  | *Lit* | *Pel* | *Lit* | *Pel* | *Lit* | *Pel* | *Lit* | *Pel* | *Lit* | *Pel* | *Lit* | *Pel* | *Lit* | *Pel* |
| **Pelagic resources** | | |  |  |  |  |  |  |  |  |  |  |  |  |  |  |
|  | *total* | | 18.9 | 16.7 | 22.7 | 427.1 | 1.4 | 107.6 | 0.8 | 1.6 | 354.6 | 67.7 | 84.5 | 77.8 | 8.4 | 606.4 |
|  | *cladocerans* | | 7.0 | 4.1 | 81.7 | 225.0 | 0.1 | 19.0 | 0.0 | 0.0 | 233.9 | 32.3 | 79.2 | 43.6 | 1.9 | 580.7 |
|  | *copepods* | | 11.8 | 12.6 | 139.0 | 202.1 | 1.4 | 88.6 | 0.8 | 1.5 | 120.7 | 35.4 | 5.2 | 34.1 | 6.5 | 25.7 |
| **Benthic resources** | | |  |  |  |  |  |  |  |  |  |  |  |  |  |  |
|  | *total* | | 1657.7 | 279.7 | 4790.8 | 258.8 | 6980.9 | 153.5 | 1426.0 | 595.7 | 1747.6 | 550.0 | 805.1 | 445.7 | 9866.4 | 22.9 |
