# Supplementary material for: Water Transparency Drives Intra-Population Divergence in Eurasian Perch (Perca fluviatilis)
Source: PLoS One. 2012 Aug 17;7(8):e43641. doi: 10.1371/journal.pone.0043641 (PMC3422328; doi:10.1371/journal.pone.0043641)
Supplement: Table S4 — Correlation matrix of predictor variables with VIP>1. Shown are Pearson’s correlation coefficients. Significance levels *p<0.05, **p<0.01. (DOCX) [file pone.0043641.s006.docx]

Table S4

|  | cf | copep | clad | doc | growth | lit roach | lit pisc | depth | secchi |
| --- | --- | --- | --- | --- | --- | --- | --- | --- | --- |
| cf | 1 | -0.59 | **0.88**** | -0.73 | 0.47 | -0.26 | 0.24 | 0.57 | **0.93**** |
| copep |  | 1 | **-0.84*** | 0.62 | -0.56 | 0.75 | -0.48 | -0.59 | -0.74 |
| clad |  |  | 1 | -0.73 | 0.61 | -0.60 | 0.41 | **0.77*** | **0.92**** |
| doc |  |  |  | 1 | -0.51 | 0.24 | -0.30 | -0.73 | **-0.82*** |
| growth |  |  |  |  | 1 | -0.52 | **0.80*** | 0.35 | 0.48 |
| lit roach |  |  |  |  |  | 1 | **-0.77*** | -0.52 | -0.49 |
| lit pisc |  |  |  |  |  |  | 1 | 0.27 | 0.39 |
| depth |  |  |  |  |  |  |  | 1 | 0.72 |
| secchi |  |  |  |  |  |  |  |  | 1 |
